# Cardiometabolic traits mediating the effect of education on osteoarthritis risk: a Mendelian randomization study

**DOI:** 10.1016/j.joca.2020.12.015

**Published:** 2021-03

**Authors:** D. Gill, V. Karhunen, R. Malik, M. Dichgans, N. Sofat

**Affiliations:** † Department of Epidemiology and Biostatistics, School of Public Health, Imperial College London, London, United Kingdom; ‡ Institute for Infection and Immunity, St George's University of London, London, United Kingdom; § St George's University Hospitals NHS Foundation Trust, London, United Kingdom; ‖ Institute for Stroke and Dementia Research (ISD), University Hospital, Ludwig-Maximilians-University (LMU), Munich, Germany; ¶ Munich Cluster for Systems Neurology (SyNergy), Munich, Germany; # German Centre for Neurodegenerative Diseases (DZNE), Munich, Germany

**Keywords:** Osteoarthritis, Education, Cardiometabolic, Mendelian randomization

## Abstract

**Objective:**

To investigate which cardiometabolic factors underlie clustering of osteoarthritis (OA) with cardiovascular disease, and the extent to which these mediate an effect of education.

**Design:**

Genome-wide association study (GWAS) of OA was performed in UK Biobank (60,800 cases and 328,251 controls) to obtain genetic association estimates for OA risk. Genetic instruments and association estimates for body mass index (BMI), low-density lipoprotein cholesterol (LDL-C), systolic blood pressure (SBP), smoking and education were obtained from existing GWAS summary data (sample sizes 188,577–866,834 individuals). Two-sample Mendelian randomization (MR) analyses were performed to investigate the effects of exposure traits on OA risk. MR mediation analyses were undertaken to investigate whether the cardiometabolic traits mediate any effect of education on OA risk.

**Results:**

MR analyses identified protective effects of higher genetically predicted education (main MR analysis odds ratio (OR) per standard deviation increase 0.59, 95% confidence interval (CI) 0.54–0.64) and LDL-C levels (OR 0.94, 95%CI 0.91–0.98) on OA risk, and unfavourable effects of higher genetically predicted BMI (OR 1.82, 95%CI 1.73–1.92) and smoking (OR 2.23, 95%CI 1.85–2.68). There was no strong evidence of an effect of genetically predicted SBP on OA risk (OR 0.98, 95% CI 0.90–1.06). The proportion of the effect of genetically predicted education mediated through genetically predicted BMI and smoking was 35% (95%CI 13–57%).

**Conclusions:**

These findings highlight education, obesity and smoking as common mechanisms underlying OA and cardiovascular disease. These risk factors represent clinical and public health targets for reducing multi-morbidity related to the burden these common conditions.

## Introduction

Osteoarthritis (OA) is the most common form of arthritis worldwide and there are currently no disease-modifying agents available. It accounts for 2.4% of all years lived with disability (YLD) and ranks as a leading contributor to global YLDs^1^. The prevalence of hip and knee OA worldwide is close to 5% and is expected to increase further^1^. Recent research on modifiable risk factors for OA have investigated the influence of cardiovascular disease and educational level. An increased prevalence of cardiovascular disease is found in OA^2^. It is also recognised that lower educational level is associated with increased cardiovascular disease^3^^,^^4^. However, the underlying mechanisms are not well understood. The effect of education on OA risk may in part be mediated cardiovascular risk factors that increase OA risk^3^^,^^4^, and evaluation of these risk factors could help to optimise disease prevention at a clinical and public health level.

Assessing causal effects in observational research is difficult due to environmental confounding or reverse causation. The Mendelian randomization (MR) approach can overcome some of these limitations by using genetic variants related to the exposure of interest as instrumental variables for investigating its effects on an outcome^5^. Genetic variants are randomly allocated at conception, and therefore their associations with the outcome are less affected by environmental confounding. More recently, MR methods have been applied to investigate mediating pathways^6^^,^^7^, where use of genetic variants that capture lifetime exposure also help overcome bias related to measurement error that can hinder observational research.

The aim of this study was to apply the MR framework to investigate the effects of education and cardiometabolic risk factors on risk of OA. For cardiometabolic risk factors that the MR analyses supported to have a causal effect on OA risk, we aimed to further apply MR mediation analyses to investigate the degree to which these factors might be mediating the effects of educational attainment.

## Methods

### Overall study design

A genome-wide association study (GWAS) for OA was performed in the UK Biobank to obtain genetic association estimates for OA risk. UK Biobank identification of OA was based on hospital-diagnosed OA cases^8^^,^^9^. Genetic association estimates for cardiometabolic cardiovascular risk factors and educational attainment (referred to hereafter as education) were selected from published GWASs. MR analyses were performed to investigate the effects of their respective genetically predicted levels on OA risk. The considered cardiometabolic risk factors were body mass index (BMI), low-density lipoprotein cholesterol (LDL-C), lifetime smoking (referred to hereafter as smoking) and systolic blood pressure (SBP). For cardiometabolic risk factors for which there was MR evidence of a detrimental effect of their genetically predicted levels on OA risk, MR mediation analyses were performed to investigate the degree to which this mediated any effect of education on OA risk.

### Osteoarthritis genome-wide association study

GWAS for OA was performed in the UK Biobank^8^^,^^9^, a prospective cohort study of approximately half a million participants with linked self-reported outcomes, health care records and genetic data. OA cases in UK Biobank were defined based on International Classification of Diseases (ICD)-9 coding (715, 721.0–721.42), ICD-10 coding (M15-M19, M47), and Office of Population Censuses and Surveys Classification of Interventions and Procedures version 4 (OPCS-4) coding (W37–W42, W52–W54, W58, W93–W95) and self-report (Supplementary Table 1 and Supplementary Fig. 1). For GWAS analysis, only white British participants defined by the UK Biobank genotyping quality control were included^8^. Baseline characteristics are provided in Table I. A total of 60,800 OA cases and 328,251 controls were included in the primary analysis. For GWAS, we used REGENIE^10^, a ridge regression based method using Firth fallback regression correcting for age, sex, the first 20 genetic principal components, genotyping chip and assessment center. In a sensitivity analysis, we also included 19,846 OA cases which were of self-report only.

**Table I:** Descriptive characteristics for the participants included in this study

Variables	Osteoarthritis (including self report)	Osteoarthritis (excluding self report)	Controls
	*N* = 80,646	*N* = 60,800	*N* = 328,250
Age, mean (SD), years	60.2 (6.7)	60.4 (6.8)	56.1 (8.1)
Sex, N (%)			
Male	33,084 (41.0)	26,073 (42.9)	154,802 (47.2)
Female	47,562 (59.0)	34,727 (57.1)	173,448 (52.8)
Never smoked, N (%)	39,876 (49.7)	29,697 (49.1)	182,523 (55.8)
Former smoker, N (%)	32,283 (40.2)	24,643 (40.7)	111,456 (34.1)
Current smoker, N (%)	8,086 (10.1)	6,149 (10.2)	33,234 (10.2)
BMI, mean (SD), kg/m^2^	28.9 (5.3)	29.1 (5.3)	27.0 (4.6)
Incident cardiovascular events, N (%)	11,410 (14.1)	9,574 (15.7)	25,185 (7.7)
Diabetes diagnosed, N (%)	5,485 (6.8)	4,432 (7.3)	14,287 (4.4)
Systolic blood pressure, mmHg (SD)	140.6 (18.4)	140.6 (18.4)	137.8 (18.7)
Diastolic blood pressure, mmHg (SD)	82.5 (9.9)	82.6 (9.9)	82.3 (10.2)

### Exposure genetic association estimates

Genetic association estimates for BMI were obtained from the GIANT Consortium GWAS meta-analysis of 806,834 European-ancestry individuals^11^. Genetic association estimates for fasting LDL-C were obtained from the Global Lipids Genetic Consortium GWAS of 188,577 European-ancestry individuals that were not taking lipid lowering medication^12^. Genetic association estimates for SBP were obtained from a GWAS of 318,417 white British individuals performed in the UK Biobank. The mean SBP from two automated recordings taken 2 min apart at baseline assessment were used, and correction for any (self-reported) anti-hypertensive medication use was made by adding 10 mmHg^3^. Genetic association estimates for smoking were obtained from a GWAS of 462,690 European-ancestry individuals in the UK Biobank^13^. A continuous lifetime smoking measure was constructed from self-reported age at initiation, age at cessation and cigarettes smoked per day^13^. Genetic association estimates for education were obtained from a GWAS of 766,345 European-ancestry individuals^14^. Education was measured as the number of years completed in full time education and was matched across different cohorts using the International Standard Classification of Education system^15^. Full details of GWAS analyses are available in their original publications.

We obtain genetic association estimates for education, BMI, SBP and smoking from studies that included UK Biobank participants^3^^,^^11^^,^^13^^,^^14^, with OA genetic association estimates also obtained from an overlapping UK Biobank population. Such participant overlap can result in bias of MR estimates towards the observational estimate in the context of weak instruments^16^. For sensitivity analysis, we conducted the analysis for education and BMI using GWAS summary statistics from non-overlapping populations^15^^,^^17^. For SBP and smoking, if evidence for association in MR was found, we estimated the potential bias due to sample overlap as previously described^16^.

### Instrument selection

Instruments for each considered exposure in univariable MR analyses were selected as single-nucleotide polymorphisms (SNPs) that associated with the exposure at genome-wide significance (*P* < 5 × 10^−8^) and were independent, i.e., pairwise linkage disequilibrium (LD) *r*^2^ < 0.001. To select instruments for multivariable MR (MVMR) in analyses investigating mediators of the effect of genetically predicted education on OA risk, all SNPs related to education or investigated mediators at genome-wide significance were pooled and clumped to pairwise LD *r*^2^ < 0.001 based on the lowest *P*-value for their association with any trait. All clumping was performed using the TwoSampleMR package in R^18^.

Genetic association estimates for different traits were aligned to correspond to the same effect allele. Palindromic variants were excluded in the main analysis and included for sensitivity analysis. Only genetic variants for which association estimates were present for all traits being studied in a given analysis were considered, and proxies were not used.

To quantify the ability to detect putative causal associations based on the available summary statistics, we calculated the minimum detectable odds ratios (OR) for the risk of OA in MR analysis of each exposure separately, given 80% power, type I error rate = 0.05, exposure GWAS summary statistics sample size and the total variance explained by the genetic instruments^19^. To evaluate instrument strength, *F* statistics were calculated for individual genetic instruments.

### Univariable Mendelian randomization

Multiplicative random-effects inverse-variance weighted (IVW) MR was used as the main analysis for estimating the effects of genetically predicted cardiovascular risk factors and education on OA risk^20^. The genetic association estimates for the OA risk were the coefficients from logistic regression (i.e., log OR) for each genetic variant. The resulting MR estimate was exponentiated to obtain the OR estimate given by MR.

When using multiple genetic variants as instrumental variables in MR, a potential source of bias is horizontal pleiotropy, where the genetic variants affect the risk of OA through pathways independent of the considered exposure. To assess the robustness of the findings to the potential bias due to horizontal pleiotropy, we used contamination-mixture method, MR-Egger and weighted median MR as sensitivity analyses^21^, ^22^, ^23^. The contamination-mixture model assumes that MR estimates from valid instruments follow a normal distribution centered on the true causal effect estimate and that those calculated from invalid instrument variants follow a normal distribution with their effect estimates centered on zero^22^. A likelihood function is then maximized for allocating each variant to one of the two mixture distributions^22^. MR-Egger performs a regression of the variant-outcome genetic association estimates on the variant-exposure genetic association estimates, weighted for the precision of the variant-outcome genetic association estimates^23^. The slope of the regression line represents the MR estimate, and evidence for directional pleiotropy can be evaluated by testing whether the intercept differs from zero^23^. In weighted median MR, the MR estimates from individual variants are ordered by their magnitude weighted for their precision, and the median is selected as the overall MR estimate, with standard errors calculated by bootstrapping^21^. The MendelianRandomization package of R was used for performing all these univariable MR analyses^24^. The discrepancy between the main IVW MR analysis and sensitivity analysis was used to assess for the potential presence of bias related to pleiotropic variants.

All MR estimates were calculated per one standard deviation (SD) unit increase in the exposure under consideration, with SD estimates derived from UK Biobank data. For BMI this was 4.77 kg/m^2^, for LDL-C this was 0.87 mmol/l, for SBP this was 18.68 mmHg and for education this was 3.6 years. For smoking, a one SD increase was equivalent to an individual smoking 20 cigarettes per day for 15 years and stopping 17 years ago, for example^13^.

### Multivariable Mendelian randomization

The genetically predicted cardiovascular risk factors that showed evidence for a detrimental effect on the risk of OA in univariable MR were taken forward for MVMR mediation analysis^7^^,^^25^. We aimed to estimate the degree to which the effect of education on the risk of OA is mediated by the cardiovascular risk factors.

In MVMR, the total effect of each exposure is decomposed to direct and indirect effects. This allows for estimation of potential mediating effects and the proportion of the effect of the main exposure of interest on the outcome that acts via other considered exposures^26^^,^^27^. Specifically, variant-OA genetic association estimates (on the log OR scale) were regressed on variant-education and variant-cardiovascular risk factor genetic association estimates, weighted for the precision (i.e., the inverse of their variance) of the variant-OA genetic association estimates and with the intercept fixed at zero^25^. The considered cardiovascular risk factors were included in this model both individually and all together. The final OR estimate of the effect of education on the risk of OA from MVMR was obtained by exponentiating the corresponding effect estimate. To estimate the proportion of the effect of genetically predicted education on OA risk that was mediated through the considered cardiovascular risk factors, the MR estimate for the effect of genetically predicted education on OA risk after adjusting for genetically predicted levels of the cardiovascular risk factors was divided by the total effect of education on OA risk estimated in the IVW univariable MR and subtracted from 1, with standard errors estimated using the propagation of error method^6^^,^^7^.

### Measuring the strength of evidence

No formal *P* value threshold for statistical significance was used. Instead, we interpret the evidence provided by the results by looking at the effect size of interest and the width of its confidence interval (CI), combined with the consistency of the results across the different methods used^28^.

### Ethical approval, data availability and reporting

All data used in this work are publicly available and the studies from which they were obtained had previously obtained relevant ethical approval and participant consent^8^^,^^9^^,^^11^^,^^12^. All data and results generated in this work are presented in the main manuscript and the related supplementary files. The reporting of this MR study follows the recommendations of the STROBE-MR Guidelines^29^, as detailed in the Supplementary Checklist. The codes for analysis are available from the authors upon request.

## Results

All genetic association estimates and their *F* statistics used in the univariable and MVMR analyses are provided in Supplementary Tables 2–9 and visualized in Supplementary Figs. 2–9. The minimum detectable ORs on the risk of OA for each outcome are given in Supplementary Table 10.

**Fig. 1:**
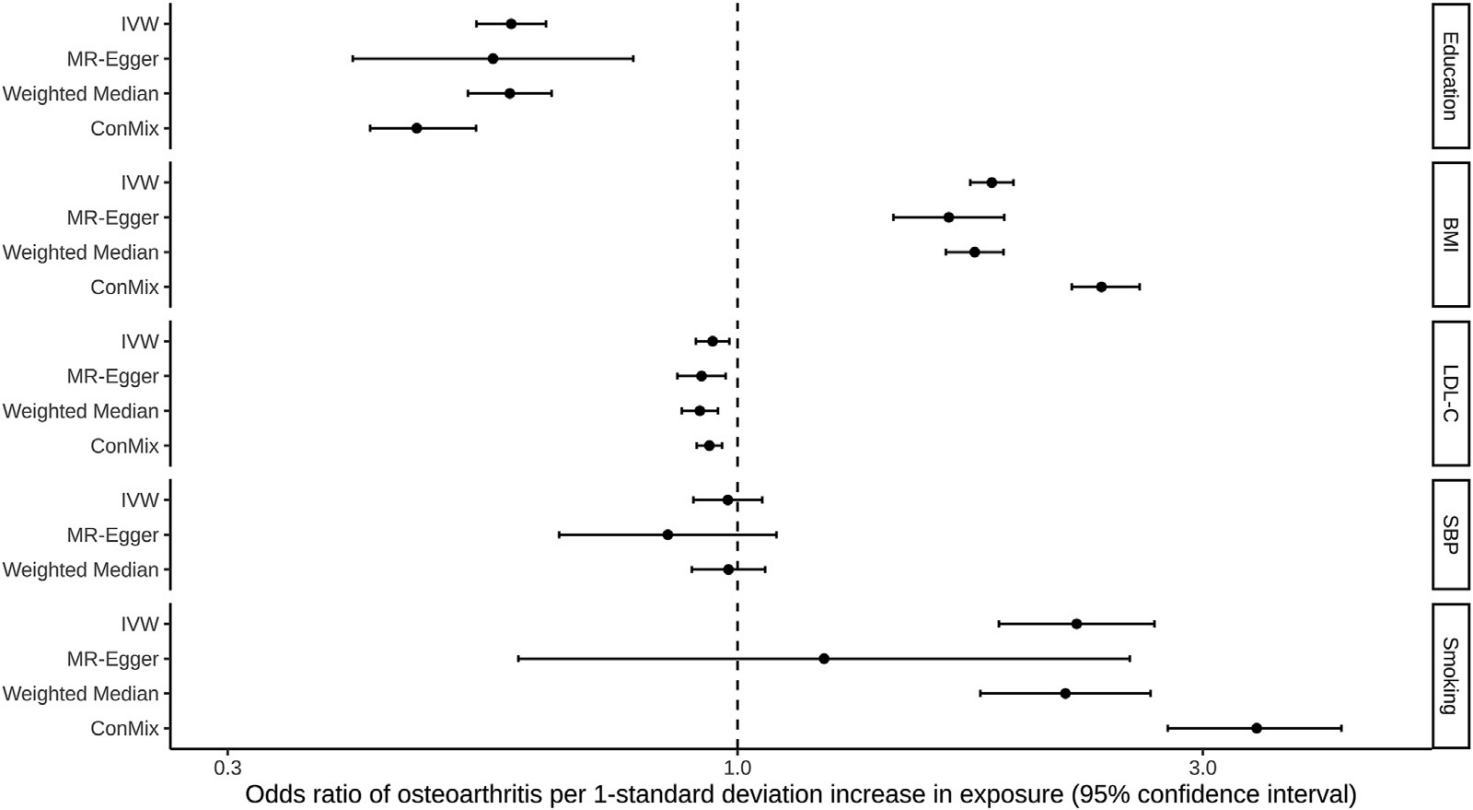
Effects of genetically predicted education, body mass index (BMI), low-density lipoprotein cholesterol (LDL-C), systolic blood pressure (SBP) and lifetime smoking respectively on risk of osteoarthritis. Inverse-variance weighted (IVW), contamination mixture (Con-Mix), Egger and weighted median represent different Mendelian randomization models. Confidence intervals could not be generated for the Con-Mix analysis considering SBP, and hence this result is not presented.

**Fig. 2:**
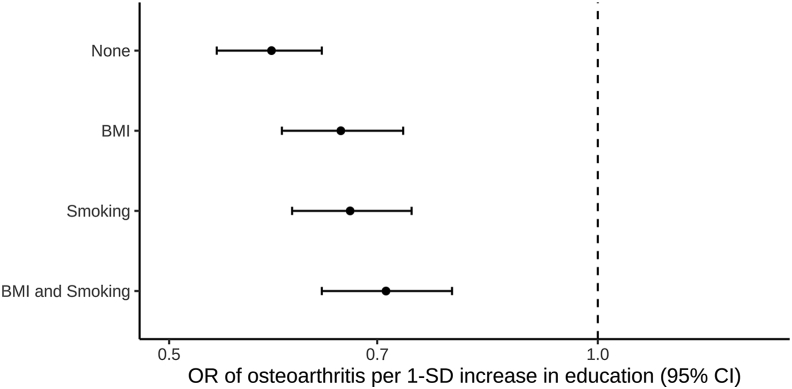
The effect of genetically predicted education on osteoarthritis risk after adjusting for genetically predicted body mass index and lifetime smoking, either separately or in the same model. The y-axis details the adjustment made. CI: confidence interval; OR: odds ratio; SD: standard deviation.

In the univariable MR, there was evidence of a protective effect of genetically predicted education and LDL-C on OA risk in the main IVW analyses (education: OR 0.59, 95% CI 0.54–0.64; LDL-C: OR 0.94, 95%CI 0.91–0.98), with consistent findings in sensitivity analyses (Fig. 1). There was evidence of an unfavourable effect of genetically predicted BMI and smoking on OA risk in the main IVW MR analyses (BMI: OR 1.82, 95%CI 1.73–1.92; smoking: OR 2.23, 95%CI 1.85–2.68), with consistent results obtained in sensitivity analyses (Fig. 1). Similar results were obtained when using non-overlapping summary statistics for BMI and education (Supplementary Table 11; Supplementary Fig. 10). The bias due to sample overlap in the log OR for smoking and the risk of OA under the null hypothesis was estimated at 0.012 and the expected Type I error rate for a two-sided test with alpha = 0.05 was estimated at 0.053. There was no evidence of an effect of genetically predicted SBP on OA risk in the main IVW (OR 0.98, 95%CI 0.90–1.06) or any MR sensitivity analysis (Fig. 1). The MR-Egger intercept tests did not give evidence for the presence of directional pleiotropy for education (*P* = 0.79), LDL-C: (*P* = 0.21), and SBP (*P* = 0.25). There was weak evidence for directional pleiotropy for BMI (*P* = 0.10) and smoking: (*P* = 0.09), however in both cases MR-Egger estimate was consistent with the IVW estimate (Fig. 1, Supplementary Table 11). Given the identified effects of higher genetically predicted BMI and higher genetically predicted smoking on increasing OA risk, MVMR mediation analyses were performed to investigate the degree to which these traits were mediating the effect of genetically predicted education on OA risk. The protective effect of genetically predicted education on OA risk attenuated from OR of 0.59 (95%CI 0.54–0.64) in IVW univariable analysis to OR of 0.66 (95%CI 0.60–0.73) after adjusting for genetically predicted BMI in MVMR analysis, to OR of 0.67 (95%CI 0.61–0.74) after adjusting for genetically predicted smoking in MVMR analysis, and to OR of 0.71 (95%CI 0.64–0.79) after adjusting for both genetically predicted BMI and genetically predicted smoking in MVMR analysis (Fig. 2).

**Fig. 3:**
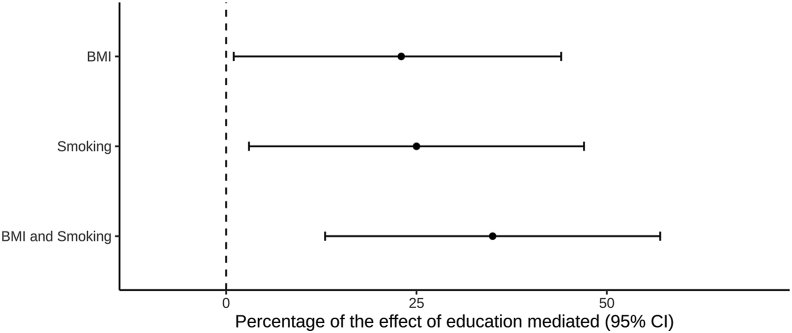
The percentage of the effect of genetically predicted education on osteoarthritis risk that is mediated through genetically predicted body mass index (BMI) and lifetime smoking, separately and when considered together in the same model. The y-axis details the mediating pathway considered. CI: confidence interval.

The proportion of the effect of genetically predicted education mediated through genetically predicted BMI, smoking, and both BMI and smoking together was estimated as 23% (95%CI 1–44%), 25% (95%CI -3%–47%) and 35% (95%CI 13–57%), respectively (Fig. 3). The results obtained by using genetic variant estimates from non-overlapping data sources showed similar directions of the mediated proportions, albeit with higher uncertainty in the estimates (Supplementary Table 12).

## Discussion

Our work uses large-scale GWAS data to investigate the effect of genetically predicted education and cardiometabolic risk factors on OA risk within the MR framework, and provides evidence supporting protective effects of education and LDL-C and unfavourable effects of BMI and smoking. These findings add insight into causal mechanisms underlying OA, its clustering with the risk factors of cardiovascular disease, and disparities related to educational attainment.

Our results are consistent with previous MR analyses identifying a protective effect of genetically predicted education and LDL-C, and a detrimental effect of genetically predicted BMI on OA risk^30^, ^31^, ^32^. However, our current study goes further to identify a novel association of genetically predicted smoking with OA risk, and additionally quantify mediation of the effect of genetically predicted education on OA risk through genetically predicted BMI and smoking. As higher education is associated with lower LDL-C^4^, this would not be consistent with LDL-C mediating the effect of education on the risk of OA and therefore LDL-C was not considered in the mediation analysis. A number of mechanisms have been proposed by which obesity and smoking might lead to increased risk and severity of OA^33^^,^^34^. In contrast to our current findings, a meta-analysis of observational studies has identified an inverse association between smoking and risk of knee OA^35^. This discrepancy may be attributable to limitations of conventional observational research for identifying causal effects^36^. Our current work also improves on a previous MR study exploring the causality of smoking on OA risk, which only incorporated a single genetic variant to proxy smoking and found an inverse association with risk of total joint replacement^37^. This discrepancy may be explained by our use of a greater number of instruments for smoking, to offer greater robustness against possible violations of the MR modelling assumptions. Furthermore, our current study also considered OA related to any joint, while the previous study only considered cases requiring hip or knee replacement^37^. As the pathophysiology of OA varies at different sites, this may also be contributing to the observed differences in findings.

The findings of our study are relevant in both clinical and public health terms. Smoking and obesity have widespread implications on human health that extend far beyond cardiovascular disease. Smoking increases risk of chronic lung disease and many cancers, while obesity is a major contributor towards risk of diabetes^38^. Targeting of these risk factors therefore represents an opportunity to simultaneously reduce risk of multiple distinct disease processes and thus ease the burden of multi-morbidity on individuals and health systems alike^38^. The identification of smoking and obesity as downstream mediators of education supports that policies intended to increase educational attainment should continue^4^^,^^39^. Educational attainment is known to be heritable, and using variants robustly associated with the trait, we were able to explore associations with OA risk. Previous work has suggested that it is the experience of being in education for longer specifically, rather than related cognitive ability, that is likely deterministic of consequent health outcomes^40^.

Our results suggest that the protective effect of education on OA risk is mediated through smoking and BMI. However, there was high uncertainty in the estimates, our data being consistent with the mediated proportion being between 13% and 57%. For comparison, approximately half of the protective effect of education on cardiovascular disease has previously been estimated to be mediated together through blood pressure, obesity and smoking^3^. Thus for OA more than cardiovascular disease, education may be having a protective effect through pathways other than downstream cardiometabolic mediators. Potential mechanisms underlying this may relate to superior self-management and healthcare engagement practices afforded to those with greater education^41^^,^^42^. Finally, our analyses also highlighted a potential protective effect of higher LDL-C levels on OA risk. However, given the small magnitude of this, and particularly in relation to the larger effect estimates seen for education, BMI and smoking (Fig. 1), it is not clear that this is of any clinical relevance.

Our study has limitations. Firstly, the MR approach uses the cumulative lifelong effect of genetic variants and should not be extrapolated to presume the effect of a clinical intervention^43^. Secondly, the possibility of reverse causation that OA causes increased BMI or liability to smoking cannot be completely ruled out. We did not examine the bidirectional associations because OA was treated as a binary phenotype, and using such binary exposure is unlikely to capture the true causal relationship in MR analysis^44^. Thirdly, the OA and smoking genetic association estimates we use were obtained using self-reported data, which may be subject to recall bias that could affect the MR estimates generated^45^. Fourthly, the UK Biobank cohort used to obtain many of the genetic association estimates in this study represents a select group that may not be representative of more general populations, and in particular non-European populations^46^^,^^47^. Fifthly, mediation analysis crucially depends on the correct formulation of the causal relationships of the exposures *a priori*, as mediation and confounding cannot be statistically distinguished^48^. We assume adult BMI and smoking mediate the effect of education, as supported by earlier literature^49^^,^^50^. Also, interpreting mediation analysis results for a binary outcome is not straightforward due to the non-collapsibility of the OR, as the estimate for the mediated proportion may be biased^7^. Finally, we considered OA at any site in these analyses, and it is possible that the determinants of OA vary across different anatomical locations^51^.

In conclusion, this study uses genetic data in MR analyses to generate evidence supporting a protective effect of education and detrimental effects of BMI and smoking on OA risk, with evidence that the effect of education is mediated through BMI and smoking. These findings highlight education, obesity and smoking as common mechanisms underlying clustering of OA with risk factors of cardiovascular disease, which may represent clinical and public health targets for reducing multi-morbidity and the burden of these common conditions.

## Author contributions

DG, and NS designed the study. DG, RM and VK performed statistical analyses. All authors interpreted the results. DG, RM and NS drafted the manuscript. All authors edited the manuscript for intellectual content. All authors take responsibility for the integrity of the study.

## Conflicts of interest

DG is employed part-time by Novo Nordisk, outside the submitted work. NS has received consultancy fees from Pfizer and Eli Lilly, but has no direct conflicts of interest relating to this project. The remaining authors have no conflicts of interest to declare.

## Funding

DG is funded by the 10.13039/100004440Wellcome Trust 4i Clinical PhD programme (Grant No. 203928/Z/16/Z) and the 10.13039/501100000274British Heart Foundation Centre of Research Excellence (RE/18/4/34215) at 10.13039/501100000761Imperial College London. NS is supported by a 10.13039/100004440Wellcome Trust Institutional Support Fund (ISSF), Grant No. 204809/Z/16/Z. This project has received funding from the European Union's Horizon 2020 research and innovation programme (666881), SVDs@target (to MD; 667375), CoSTREAM (to MD); the DFG as part of the Munich Cluster for Systems Neurology (SyNergy, EXC EXC 2145 SyNergy – ID 390857198), the CRC 1123 (B3; to MD) and project DI 722/13-1; the Corona Foundation (to MD); the LMUexcellent fond (to MD); the e:Med program (e:AtheroSysMed; to MD) and the FP7/2007–2103 European Union project CVgenes@target (grant agreement number Health-F2-2013-601456; to MD). The funding sources were not involved in study design, acquisition of data, analysis, interpretation or manuscript write up.
