# Supplementary material for: Cardiometabolic traits mediating the effect of education on osteoarthritis risk: a Mendelian randomization study
Source: Osteoarthritis Cartilage. 2021 Mar;29(3):365–71. doi: 10.1016/j.joca.2020.12.015 (PMC7955282; doi:10.1016/j.joca.2020.12.015)
Supplement: Multimedia component 1 [file mmc1.docx]

**Supplementary Material**

# **Cardiometabolic traits mediating the effect of education on osteoarthritis risk: a Mendelian randomization study**

| ICD10 | |  | OPCS4 | |  | ICD9 | |
| --- | --- | --- | --- | --- | --- | --- | --- |
| Code | N |  | Code | N |  | Code | N |
| M15 | 5,461 |  | W37 | 6,714 |  | 715 | 156 |
| M16 | 14,713 |  | W38 | 5,396 |  | 721.0-721.42 | 34 |
| M17 | 24,279 |  | W39 | 1,303 |  |  |  |
| M18 | 1,694 |  | W93 | 517 |  |  |  |
| M19 | 21,728 |  | W94 | 1,974 |  |  |  |
| M47 | 9,884 |  | W95 | 154 |  |  |  |
|  |  |  | W58 | 4,009 |  |  |  |
|  |  |  | W40 | 11,521 |  |  |  |
|  |  |  | W41 | 906 |  |  |  |
|  |  |  | W42 | 1,047 |  |  |  |
|  |  |  | W58 | 4,009 |  |  |  |
|  |  |  | W52 | 647 |  |  |  |
|  |  |  | W53 | 98 |  |  |  |
|  |  |  | W54 | 229 |  |  |  |

Supplementary Table 1. The number of cases per each diagnosis code in ICD-10, OPCS4 and ICD-9.

Supplementary Table 2. Genetic variants used for Mendelian Randomization analysis of the effect of BMI on the risk of OA. CHR = chromosome; POS = position; EA = effect allele; NEA = non-effect allele; BETA = effect size estimate; SE = standard error. See separate Excel file.

Supplementary Table 3. Genetic variants used for Mendelian Randomization analysis of the effect of education on the risk of OA. CHR = chromosome; POS = position; EA = effect allele; NEA = non-effect allele; BETA = effect size estimate; SE = standard error. See separate Excel file.

Supplementary Table 4. Genetic variants used for Mendelian Randomization analysis of the effect of LDL-C on the risk of OA. CHR = chromosome; POS = position; EA = effect allele; NEA = non-effect allele; BETA = effect size estimate; SE = standard error. See separate Excel file.

Supplementary Table 5. Genetic variants used for Mendelian Randomization analysis of the effect of systolic blood pressure on the risk of OA. CHR = chromosome; POS = position; EA = effect allele; NEA = non-effect allele; BETA = effect size estimate; SE = standard error. See separate Excel file.

Supplementary Table 6. Genetic variants used for Mendelian Randomization analysis of the effect of smoking on the risk of OA. CHR = chromosome; POS = position; EA = effect allele; NEA = non-effect allele; BETA = effect size estimate; SE = standard error. See separate Excel file.

Supplementary Table 7. Genetic variants used for multivariable Mendelian Randomization analysis of the effect of education and BMI on the risk of OA. CHR = chromosome; POS = position; EA = effect allele; NEA = non-effect allele; BETA = effect size estimate; SE = standard error. See separate Excel file.

Supplementary Table 8. Genetic variants used for multivariable Mendelian Randomization analysis of the effect of education and smoking on the risk of OA. CHR = chromosome; POS = position; EA = effect allele; NEA = non-effect allele; BETA = effect size estimate; SE = standard error. See separate Excel file.

Supplementary Table 9. Genetic variants used for multivariable Mendelian Randomization analysis of the effect of education, BMI and smoking on the risk of OA. CHR = chromosome; POS = position; EA = effect allele; NEA = non-effect allele; BETA = effect size estimate; SE = standard error. See separate Excel file.

|  |  |  |  | F statistic | |
| --- | --- | --- | --- | --- | --- |
| Exposure | Outcome | Variance explained | Minimum detectable odds ratio^a^ | Median | Total |
| Education | OA | 0.022 | 1.09 | 45 | 16,933 |
| BMI | OA | 0.056 | 1.05 | 60 | 45,055 |
| LDL-C | OA | 0.098 | 1.04 | 87 | 18,548 |
| SBP | OA | 0.029 | 1.08 | 37 | 9,275 |
| Smoking | OA | 0.005 | 1.19 | 17 | 2,472 |

Supplementary Table 10. Minimum detectable odds ratios, median F statistic and the sum of F statistics across all genetic variants used as instruments for MR analysis with OA as the outcome. ^a^With power = 0.8 and type I error rate = 0.05.

Supplementary Table 11. All univariable Mendelian Randomization results. Note that the contamination mixture method does not produce standard errors. BETA = effect size estimate; SE = standard error; OR = odds ratio; CIL = 95% confidence interval, lower limit; CIU = 95% confidence interval, upper limit; IVW-RE = Multiplicative random-effects inverse-variance weighted method; ConMix = contamination mixture method. See separate Excel file.

Supplementary Table 12. Multivariable Mendelian Randomization results with different with and without using exposure summary statistics from overlapping studies (UK Biobank). OR = odds ratio; CI = confidence interval.

|  | | | Effect of education in univariable MR | Effect of education in multivariable MR |  |
| --- | --- | --- | --- | --- | --- |
| Exposures | Outcome | UK Biobank included in exposure | OR (95% CI) | OR (95% CI) | Estimated proportion mediated (95% CI) |
| Education, BMI | OA | Yes | 0.59 (0.54 to 0.64) | 0.66 (0.60 to 0.73) | 23% (1% to 44%) |
|  |  | No | 0.61 (0.53 to 0.71) | 0.65 (0.56 to 0.75) | 13% (-27% to 52%) |
| Education, smoking | OA | Yes | 0.59 (0.54 to 0.64) | 0.67 (0.61 to 0.74) | 25% (3% to 47%) |
|  |  | No | 0.61 (0.53 to 0.71) | 0.69 (0.58 to 0.82) | 24% (-18% to 65%) |
| Education, BMI, smoking | OA | Yes | 0.59 (0.54 to 0.64) | 0.71 (0.64 to 0.79) | 35% (13% to 57%) |
|  |  | No | 0.61 (0.53 to 0.71) | 0.74 (0.63 to 0.87) | 39% (1% to 77%) |


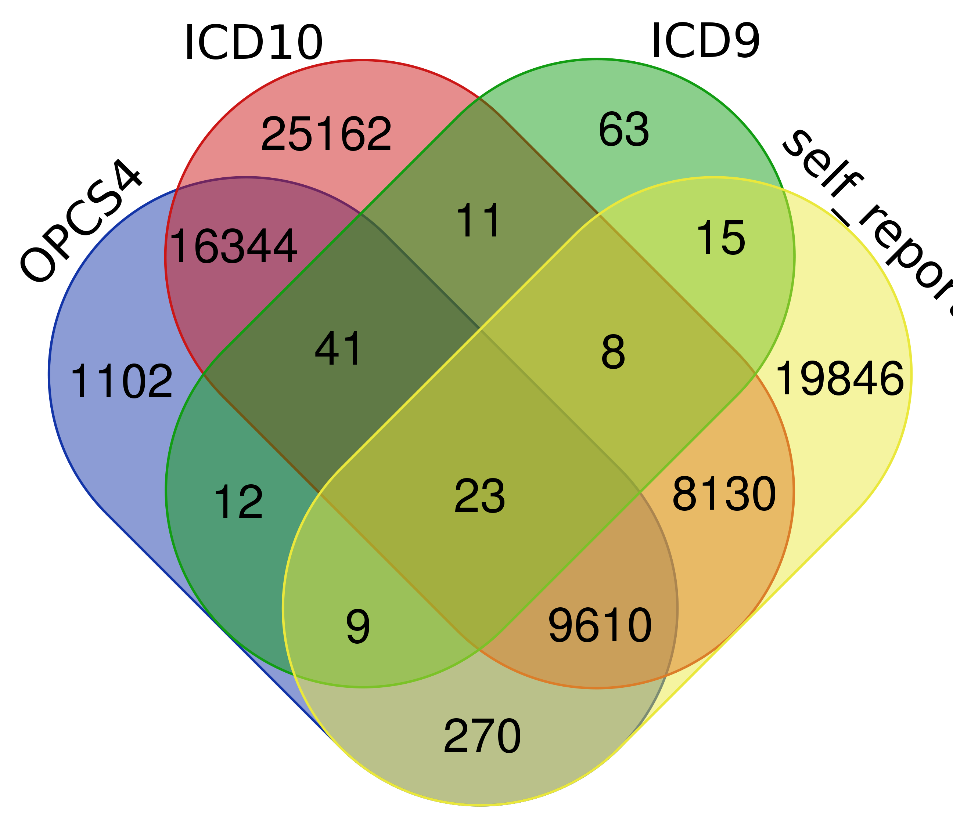


Supplementary Figure 1. Breakdown of osteoarthritis cases according to different sources.


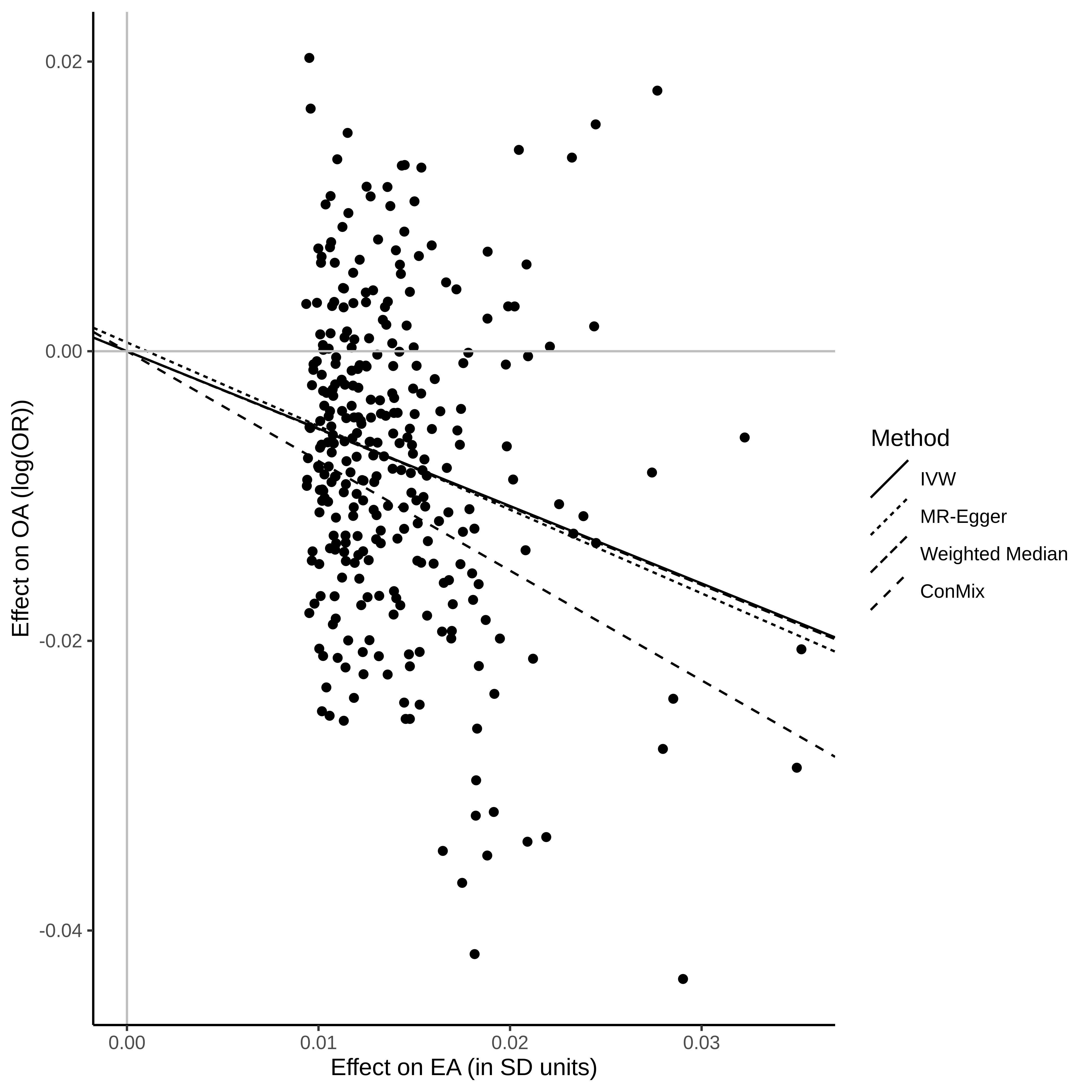


Supplementary Figure 2. A scatterplot of variant-education (x-axis) and variant-OA risk (log-odds, y-axis) association estimates for education instruments in univariable MR analysis, with lines added for MR results using different methods. The slope of each line corresponds to the effect size obtained by the corresponding MR method.


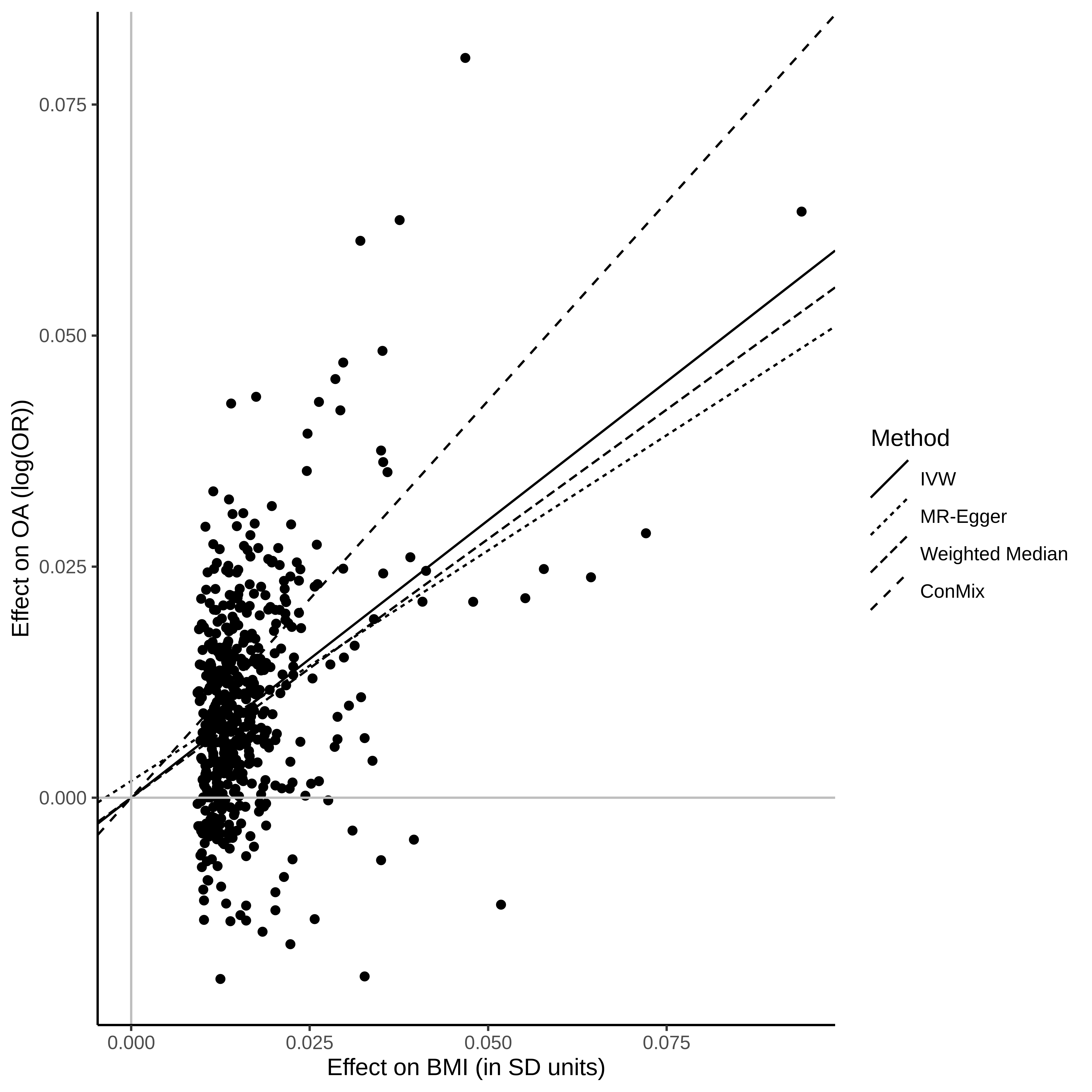


Supplementary Figure 3. A scatterplot of variant-BMI (x-axis) and variant-OA risk (log-odds, y-axis) association estimates for BMI instruments in univariable MR analysis, with lines added for MR results using different methods. The slope of each line corresponds to the effect size obtained by the corresponding MR method.


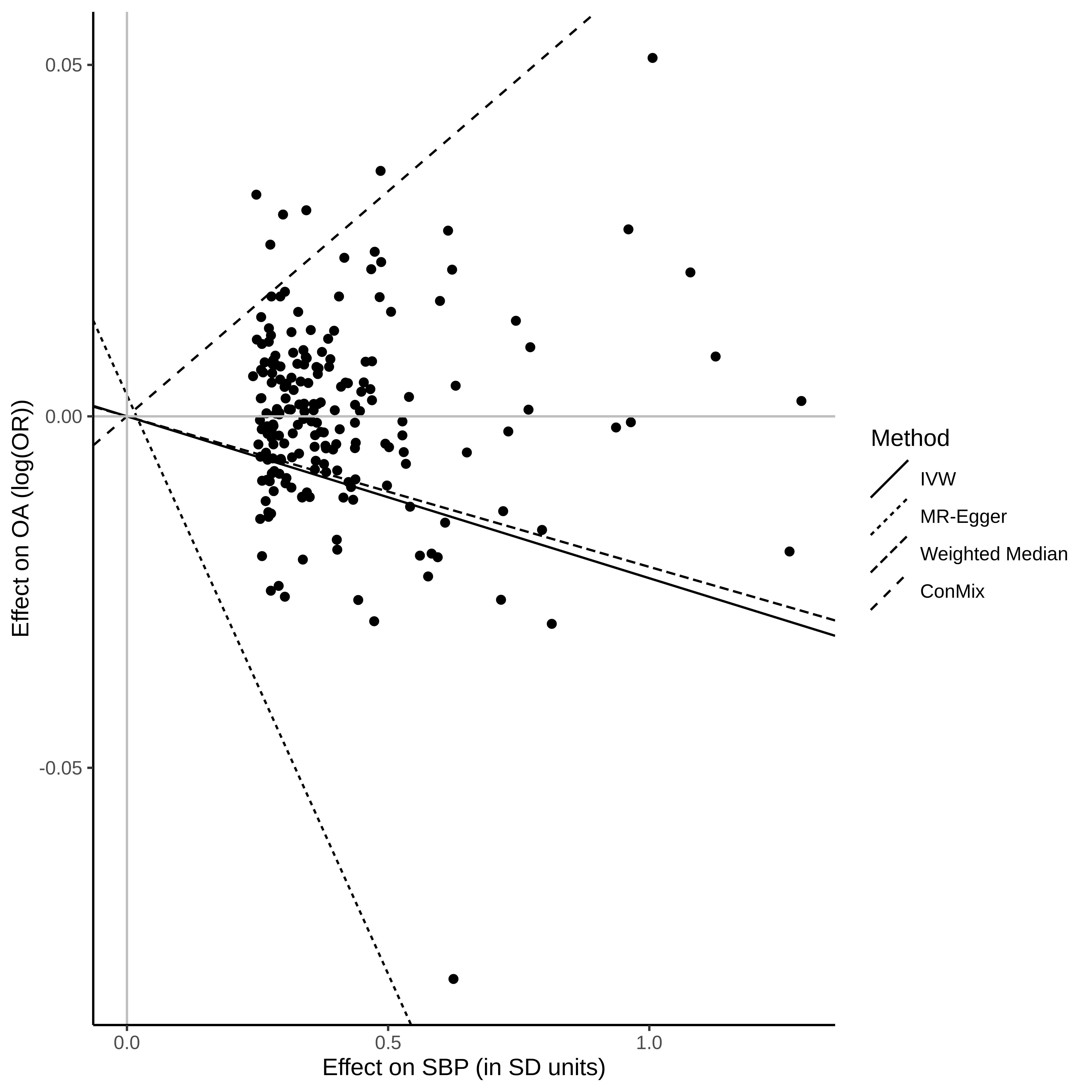
Supplementary Figure 4. A scatterplot of variant-SBP (x-axis) and variant-OA risk (log-odds, y-axis) association estimates for SBP instruments in univariable MR analysis, with lines added for MR results using different methods. The slope of each line corresponds to the effect size obtained by the corresponding MR method.


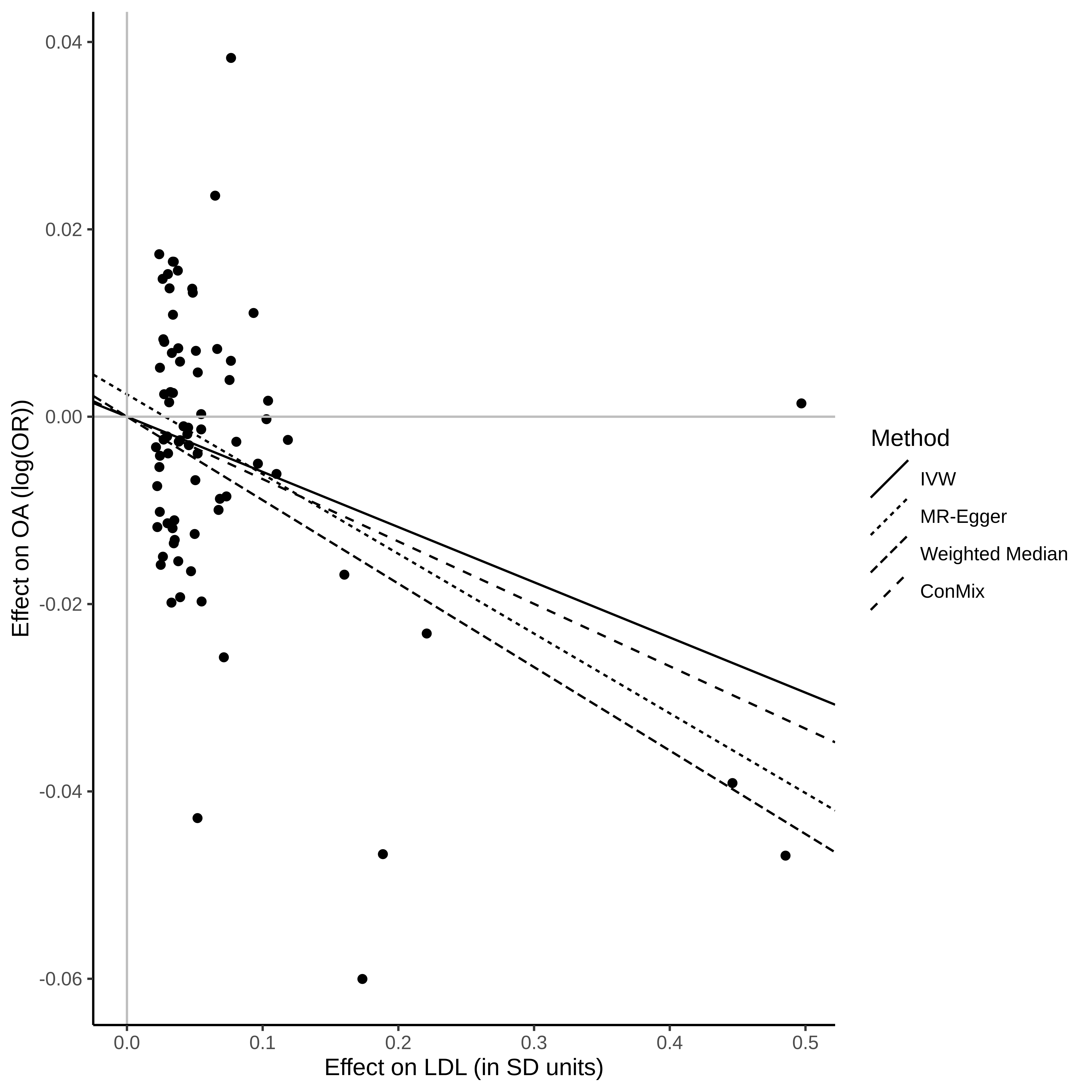


Supplementary Figure 5. A scatterplot of variant-LDL-C (x-axis) and variant-OA risk (log-odds, y-axis) association estimates for LDL-C instruments in univariable MR analysis, with lines added for MR results using different methods. The slope of each line corresponds to the effect size obtained by the corresponding MR method.


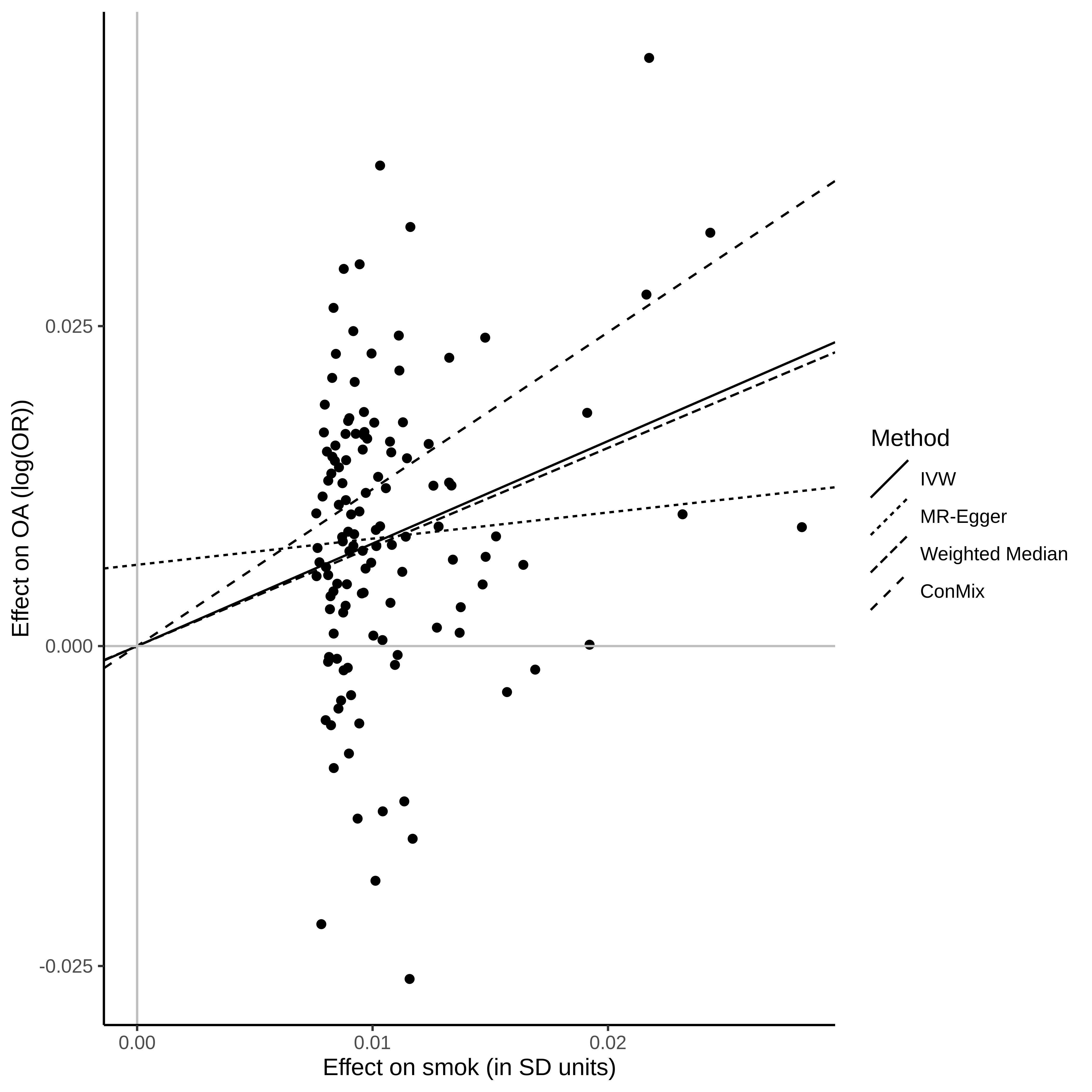


Supplementary Figure 6. A scatterplot of variant-smoking (x-axis) and variant-OA risk (log-odds, y-axis) association estimates for smoking instruments in univariable MR analysis, with lines added for MR results using different methods. The slope of each line corresponds to the effect size obtained by the corresponding MR method.


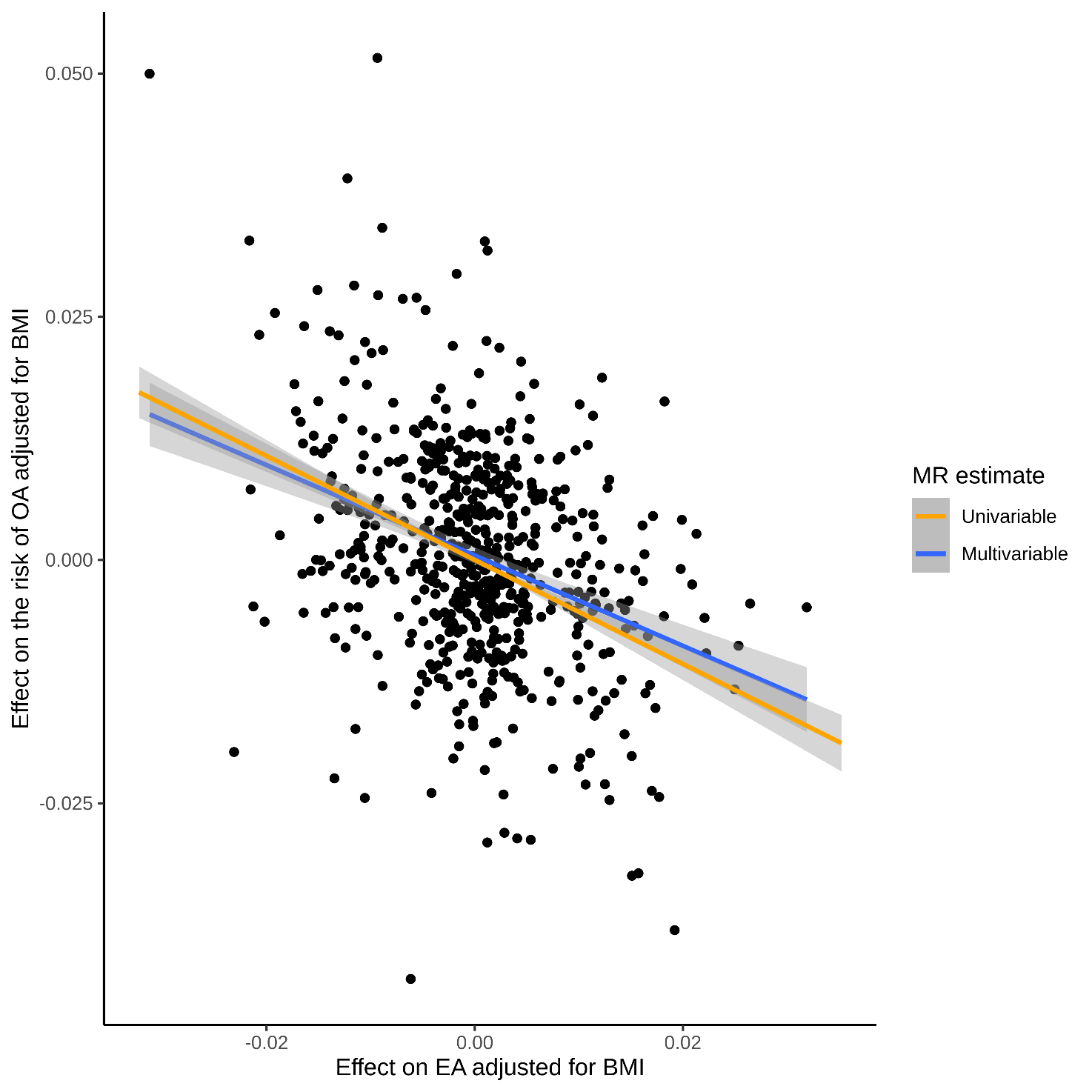


Supplementary Figure 7. Added variable plot for the effect of education on the risk of OA, adjusted for BMI, in multivariable MR. X-axis is the residuals from inverse-variance weighted MR of BMI on EA, i.e. residuals from regressing variant-education estimates on variant-BMI estimates with intercept at zero, weighted by precision of variant-education estimates. Y-axis is the residuals from inverse-variance weighted MR of BMI on the risk of OA, i.e. residuals from regressing (log-odds) variant-OA estimates on variant-BMI estimates with intercept at zero, weighted by precision of variant-education estimates. The slope of the scatterplot (with intercept fixed at zero and weighted by the precision of variant-OA estimates) is the multivariable MR effect size of education on the risk of OA, adjusted for BMI. The univariable MR effect size estimate of education on the risk of OA is added for comparison. The shaded regions represent 95% confidence intervals.


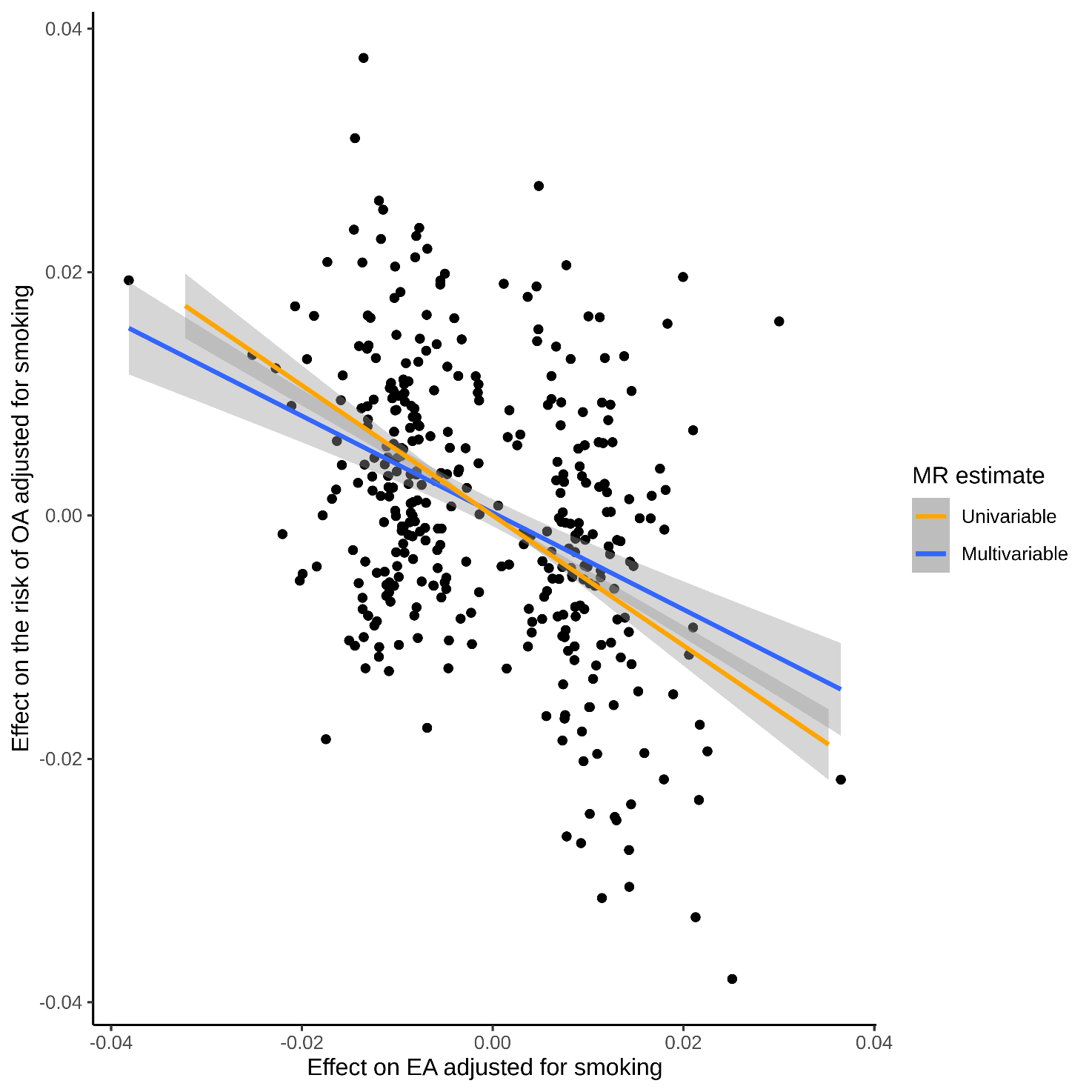


Supplementary Figure 8. Added variable plot for the effect of education on the risk of OA, adjusted for smoking, in multivariable MR. X-axis is the residuals from inverse-variance weighted MR of smoking on EA, i.e. residuals from regressing variant-education estimates on variant-smoking estimates with intercept at zero, weighted by precision of variant-education estimates. Y-axis is the residuals from inverse-variance weighted MR of smoking on the risk of OA, i.e. residuals from regressing (log-odds) variant-OA estimates on variant-smoking estimates with intercept at zero, weighted by precision of variant-education estimates. The slope of the scatterplot (with intercept fixed at zero and weighted by the precision of variant-OA estimates) is the multivariable MR effect size of education on the risk of OA, adjusted for smoking. The univariable MR effect size estimate of education on the risk of OA is added for comparison. The shaded regions represent 95% confidence intervals.


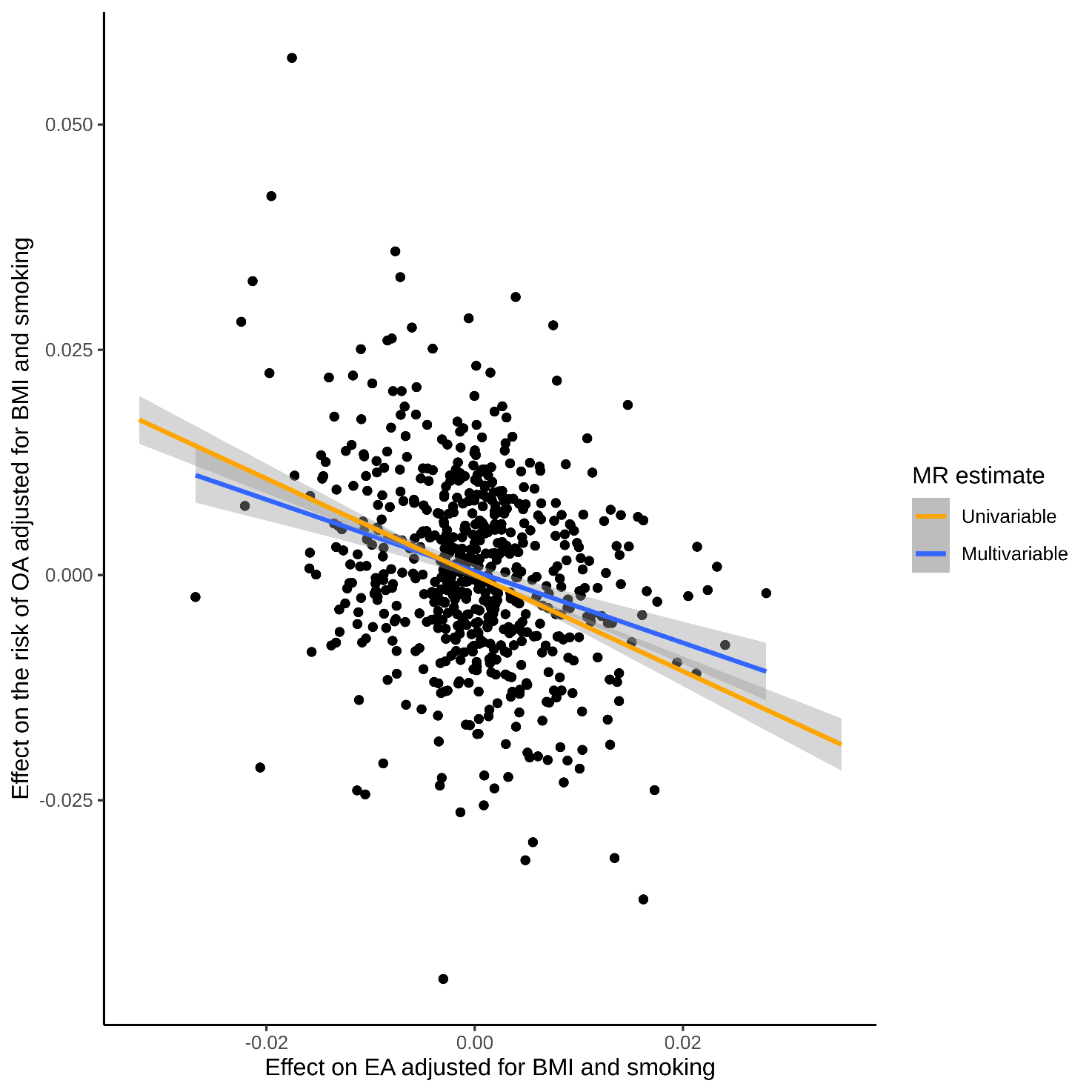


Supplementary Figure 9. Added variable plot for the effect of education on the risk of OA, adjusted for BMI and smoking, in multivariable MR. X-axis is the residuals from inverse-variance weighted MR of BMI and smoking on EA, i.e. residuals from regressing variant-education estimates on variant-BMI and variant-smoking estimates with intercept at zero, weighted by precision of variant-education estimates. Y-axis is the residuals from inverse-variance weighted MR of BMI and smoking on the risk of OA, i.e. residuals from regressing (log-odds) variant-OA estimates on variant-BMI and variant-smoking estimates with intercept at zero, weighted by precision of variant-education estimates. The slope of the scatterplot (with intercept fixed at zero and weighted by the precision of variant-OA estimates) is the multivariable MR effect size of education on the risk of OA, adjusted for BMI and smoking. The univariable MR effect size estimate of education on the risk of OA is added for comparison. The shaded regions represent 95% confidence intervals.


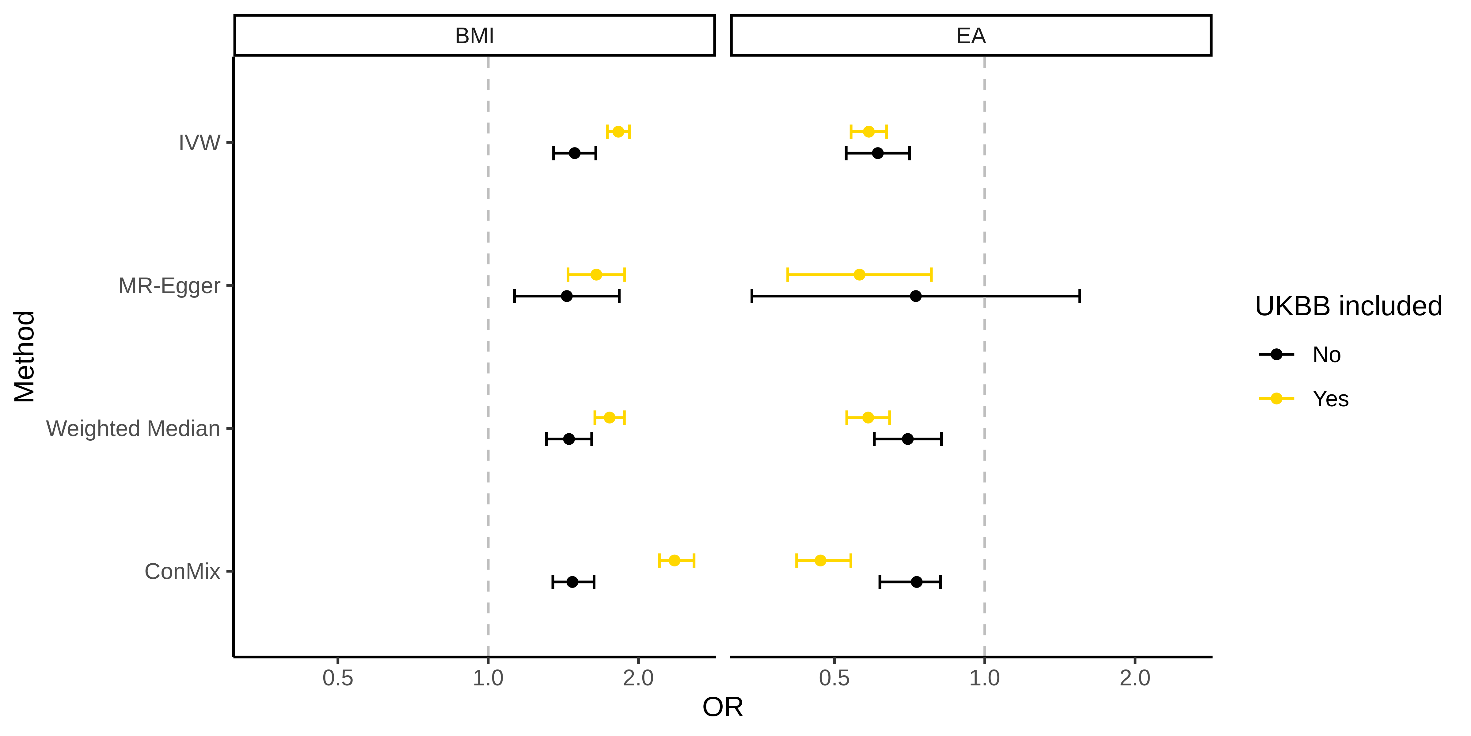


Supplementary Figure 10. Comparison of Mendelian Randomization (MR) results with the risk of osteoarthritis as the outcome for datasets including/excluding UK Biobank (UKBB) participants. BMI = body mass index; EA = education; OR = Odds ratio; IVW = inverse variance weighted method; ConMix = contamination mixture method.
